# Antagonist Properties of *Conus parius* Peptides on N-Methyl-D-Aspartate Receptors and Their Effects on CREB Signaling

**DOI:** 10.1371/journal.pone.0081405

**Published:** 2013-11-18

**Authors:** Shailaja Kunda, John Cheriyan, Michael Hur, Rashna D. Balsara, Francis J. Castellino

**Affiliations:** W.M. Keck Center for Transgene Research and Department of Chemistry and Biochemistry, University of Notre Dame, Notre Dame, Indiana, United States of America; University of Edinburgh, United Kingdom

## Abstract

Three members of a family of small neurotoxic peptides from the venom of *Conus parius*, conantokins (Con) Pr1, Pr2, and Pr3, function as antagonists of N-methyl-D-aspartate receptors (NMDAR). We report structural characterizations of these synthetic peptides, and also demonstrate their antagonistic properties toward ion flow through NMDAR ion channels in primary neurons. ConPr1 and ConPr2 displayed moderate increases in α-helicity after addition of Mg^2+^. Native apo-ConPr3 possessed an α-helical conformation, and the helicity increased only slightly on addition of Mg^2+^. Additionally, these peptides diminished NMDA/Gly-mediated currents and intracellular Ca^2+^ (iCa^2+^) influx in mature rat primary hippocampal neurons. Electrophysiological data showed that these peptides displayed slower antagonistic properties toward the NMDAR than conantokins from other species of cone snails, e.g., ConT and ConG. Furthermore, to demonstrate selectivity of the *C. parius*-derived conantokins towards specific NMDAR subunits, cortical neurons from GluN2A^-/-^ and GluN2B^-/-^ mice were utilized. Robust inhibition of NMDAR-mediated stimulation in GluN2A^-/-^-derived mouse neurons, as compared to those isolated from GluN2B^-/-^-mouse brains, was observed, suggesting a greater selectivity of these antagonists towards the GluN2B subunit. These *C. parius* conantokins mildly inhibited NMDAR-induced phosphorylation of CREB at Ser^133^, suggesting that the peptides modulated iCa^2+^ entry and, thereby, activation of CREB, a transcription factor that is required for maintaining long-term synaptic activity. Our data mechanistically show that while these peptides effectively antagonize NMDAR-directed current and iCa^2+^ influx, receptor-coupled CREB signaling is maintained. The consequence of sustained CREB signaling is improved neuronal plasticity and survival during neuropathologies.

## Introduction

The conantokins are a diverse array of 17-27-amino acid residue peptides found in the venoms of marine snails of the genus *Conus* that aid the host in the capture of prey and enable their defense against predators [1]. The first conantokin identified, conantokin-G (ConG), was purified and characterized from *Conus geographus* [2]. Later a variety of conantokins, such as ConT [3] and ConR [4], were discovered and characterized from other hunting snail species. Notably, these peptides have multiple conserved *gamma*-carboxyglutamate (Gla) residues that are central to their biological activity, *viz*., selective inhibition of ion flow through the Glu/Gly co-agonized channels of the N-methyl-D-aspartate receptor (NMDAR) [2]. Additionally, the Gla residues give rise to a rigid conformation of the peptides, especially in the presence of divalent cations, which stabilizes an α-helical conformation of the conantokins, and allows for peptide dimerization [5]. However, the extent of α-helical conformation varies between these peptides. ConT assumes a high degree of α-helicity in the metal-free (apo) state, whereas ConG adopts a random conformation in absence of divalent ions [6,7]. However, ConG binds to divalent cations to generate an α-helical structure comparable to apo- or metal-bound ConT [8].

Since the first conantokin was reported over two decades ago, these peptides have generated interest as selective antagonists of the NMDAR. The Glu/Gly co-activated NMDAR is a voltage- and ligand-gated ion channel, which plays an important role in excitatory synaptic transmission, with ramifications in plasticity, learning, and memory. The functional NMDAR of the Central Nervous System (CNS) is a heterotetramer composed of two classes of subunits. The Gly-binding GluN1 subunit is ubiquitously expressed and belongs to one of its eight splice variants, GluN1a-GluN1h. This co-assembles with a Glu binding GluN2 subunit, the expression of which varies spatially and temporally [9]. The GluN2 family consists of four different subunits, GluN2A-GluN2D, encoded by separate genes [10]. The exact subunit composition of the NMDAR determines differences in binding of agonists and antagonists [11]. Although NMDARs are essential for mediating development and normal synaptic transmission, they are excessively activated in many neuropathologies [12,13]. For example, reports have demonstrated the involvement of the GluN2B subunit of an NMDAR subset in Huntington’s disease [14], epileptogenesis [15], and opiate addiction [16], among others. Therefore, GluN2B-specific antagonistic activity is a much-sought property as neuroprotective drugs, as opposed to less desirable high affinity NMDAR ion channel blockers [17]. Toward this end, the GluN2B-specificity of ConG has been exploited as an anti-convulsive and an analgesic agent [18,19], as well as a neuroprotective agent for post-ischemic injury [20,21].

Recently, other conantokins were discovered in the venom of the Indo-Pacific fish hunting cone snail, *C. parius*. These peptides, identified as ConPr1, ConPr2, and ConPr3, differ in several residues from other Gla-containing conantokins previously reported, such as the absence of Gla at position 3, and the presence of three different post-translational modifications in ConPr3, *viz*., Gla residues, a hydroxyproline residue at position 3, and C-terminal amidation [22]. Despite these differences, these peptides exhibited high inhibitory potency for GluN2B-containing NMDARs, which were transfected into *Xenopus* oocytes [22]. However, the specificities and efficacies of these peptides, and their effects on signaling downstream of the NMDAR, were not tested in physiologically-relevant neuronal cells that contain a complex system of dynamic receptors. In the past, we have documented that the inhibition by ConG, ConT, and ConR of NMDA/Gly-stimulated intracellular Ca^2+^ (iCa^2+^) influx affected downstream activation of the transcription factor, cAMP response element-binding protein (CREB) at Ser^133^, a property that depended on neuron maturity [23].

In the current study, the *ex vivo* biological activity of this new family of peptides has been evaluated by performing whole cell patch clamp current recordings in mature primary neurons derived from wild-type (WT), GluN2A^-/-^, and GluN2B^-/-^ mouse cortices, and those from the rat hippocampus. Selectivity towards NMDAR subunits was measured through real-time changes in iCa^2+^. Since Ca^2+^ influx is directly linked to downstream signaling activities, this study also afforded an understanding of the effects of these particular peptides on CREB signaling. 

## Materials and Methods

### Animal studies

Control C57Bl/6 mice were obtained from Jackson Laboratories (Bar Harbor, ME). The previously described GluN2A^-/-^ mice [24] were provided by Dr. Gary Westbrook, Oregon Health and Science University. GluN2B^-/-^ mice [25] were obtained from National Institutes of Health/National Institute of Alcohol Abuse and Alcoholism. Both, GluN2A^-/-^ and GluN2B^-/-^ mice were fully backcrossed in the C57Bl/6 background. 

Sprague-Dawley rats and mice of breeding age (at least 8 weeks of age), with timed pregnancies of 17.5-18.5 days gestation, were used for these studies. At this time, the individual was placed in a CO_2_ chamber with a flow rate set to displace 16% of the cage volume/min. The gas was administered until asphyxiation occurred. The animal was then decapitated and fetuses immediately removed under sterile conditions. The head of each fetus was severed and placed into Hibernate E medium (BrainBits, Life Technologies, Springfield, IL). The brains were excised and the hippocampus region (rat) or cortical region (mouse) was isolated and processed for obtaining neuronal cultures (University of Notre Dame IACUC approved protocol 14-086). 

### Conantokin synthesis

The following conantokins were chemically synthesized as described earlier [8], where γ refers to γ-carboxyglutamate:

ConPr1: GEDγYAγGIRγYQLIHGKI
ConPr2: DEOγYAγAIRγYQLKYGKI
ConPr3: GEOγVAKWAγGLRγKASSN-CONH_2_

Scrambled peptide: GIKAQRDILYYγGγGγEHI-CONH_2_. This scrambled peptide, based on the sequence of ConPr1, was designed using an online scrambled peptide generator program (www.mimotopes.com, Mimotopes, Clayton, Victoria, Australia) and was employed in this work as a negative control. 

The conantokins were synthesized using standard N-(9-fluorenyl)methoxycarbonyl (Fmoc)-protected amino acids (Novabiochem, La Jolla, CA) by solid phase peptide synthesis using a Model 433A Applied Biosystems peptide synthesizer (Foster City, CA). The standard 0.1 mmol scale of synthesis was carried out, wherein a 10X excess of amino acids and a 5X excess of Fmoc-di(tBu)-Gla was used [26]. After synthesis, the peptide was cleaved from the resin by treatment with 10 ml of a mixture of trifluoroacetic acid/triisopropylsilane/dithiothreitol/water (88:2:5:5 volume ratios) with gentle stirring for 3 hr. The mixture was filtered and reduced to a constant volume. Ice-cold diethyl ether (40 ml) was added to facilitate precipitation. The lyophilized crude peptide was dissolved in 5 ml water and filtered though a Sephadex G-15 (Sigma, St. Louis, MO) column, using 0.1% acetic acid or NH_4_OH as running solutions depending on the pI of the peptide. The purities of the peptides were determined by analytical HPLC (Beckman Coulter, Brea, CA) using a Vydac C18 analytical column (Resolution Systems, Holland, MI), as well as by MALDI-TOF (Bruker Daltonics, Fremont, CA). If required, further purification of the conantokins was carried out by ion exchange chromatography. 

### Circular dichroism (CD)

CD spectra were recorded on an AVIV (Lakewood, NJ) 202SF spectrometer. Spectral measurements were obtained at room temperature in a 0.1 cm path length quartz cuvette at a wavelength range of 200-250 nm. Scans were collected at 1.0 nm intervals at a 1.0 nm bandwidth. MgCl_2_ (2 mM) was added and allowed to equilibrate with the peptide for 2 hr. The molar ellipticity (θ, radians) was calculated from; θ = [100 × (ΔA_222nm_)/(*n*–1) × *L* × (90 µM peptide)] where *n* = number of residues in the peptide; *L* = path-length of the cuvette in cm; and ΔA_222nm_ is the CD signal (mdeg at 222 nm).  

### Cell cultures of dissociated primary neurons

Primary rat hippocampal neuron cultures were prepared from embryonic day (ED)-18 Sprague-Dawley rat embryos as previously described [23]. Neuronal cultures prepared from such late stage embryos are known to yield a homogenous population of hippocampal pyramidal cells with low levels of contamination by glial cells [27].

Neurons were dissociated using 2 mg/ml papain and plated on 14 mm glass-bottom microwell dishes (Mat Tek, Ashland, MA) or 35 mm tissue culture-treated dishes (Corning Life Sciences, Lowell, MA) coated with poly-L-lysine in Neurobasal medium (Invitrogen, Carlsbad, CA) supplemented with 2% B27 (Invitrogen)/1% L-Glu. Cell cultures were maintained at 37° C in a humidified atmosphere with 5% CO_2_. Cortical neurons were dissociated with 1 mg/ml papain from ED-18 embryos of WT, GluN2A^-/-^, and GluN2B^-/-^ mice. To obtain GluN2B^-/-^ embryos, mice heterozygous for the GluN2B allele were bred and embryos at ED-18 were harvested and genotyped for the double GluN2B^-/-^ alleles. GluN2B^-/-^ embryos were used for neuron culture.

### Calcium imaging

Rat hippocampal and mouse cortical neurons were seeded at a density of 2.5 x 10^5^ cells/ml on poly-L-lysine-coated 14 mm glass-bottom microwell dishes (Mat Tek), washed 3X with ACSF (artificial cerebrospinal fluid: 140 mM NaCl/5 mM KCl/2 mM CaCl_2_/10 mM HEPES/24 mM glucose, pH 7.2), and incubated with 1 μM fura-2-acetoxymethyl ester (Fura-2/AM, Invitrogen) at room temperature for 30 min. After this time, the cells were washed 3X with ACSF and the neurons were incubated for 15–30 min in the same solution to allow de-esterification of intracellular AM esters. The dish was mounted onto an imaging chamber and placed on the stage of a Nikon Eclipse TE 2000-S microscope (Nikon Instruments, Melville, NY). Neuron bodies were marked as regions of interest using the tool of the NIS-Elements AR 3.0 software program (Nikon). Application of ACSF, or stimulation with 50 μM NMDA/10 μM Gly ± conantokin, was performed using a ValveBankII perfusion system (AutoMate Scientific, Berkeley, CA) at a manually controlled flow rate of 1 ml/min. The neurons were exposed to alternating 340 nm and 380 nm light from a xenon lamp *via* a shutter (Sutter Instruments, Novato, CA). The resulting images were captured with a Cascade II 512 camera (Photometrics, Tucson, AZ) and acquired at 2 sec intervals for 60 sec before stimulation to obtain a steady baseline. Next, the neurons were stimulated with 50 μM NMDA/10 μM Gly until a plateau in signal was reached. After this, the cells were washed with ACSF, then preincubated with either 5 or 10 μM ConPr1, ConPr2, or ConPr3 for 3 min, and subsequently re-stimulated with 50 μM NMDA/10 μM Gly. Control experiments were performed wherein neurons were re-stimulated with a mixture of 50 μM NMDA/10 μM Gly with either 5 μM scrambled peptide, 60 nM NVP-AAM077 (Gift from Y.P. Auberson, Novartis, Basel, Switzerland), 400 nM NVP-AAM077, or 3 μM ifenprodil. Ratiometric traces were generated using NIS-Elements software. The iCa^2+^ influx stimulated by NMDA/Gly, before and after pre-incubation with conantokins, was calculated by subtracting the basal value from the peak value and plotted as increase above basal Ca^2+^ levels. Changes in iCa^2+^ responses induced by NMDA/Gly alone were then compared to responses elicited by NMDA/Gly after the neurons were exposed to conantokins. These changes in iCa^2+^ levels were reported as mean ± S.E.M from at least three independent sample sets for conantokins and the control experiments were performed on one or two independent sample sets of rat hippocampal neurons, where the data reported are an average of at least 8 neurons.

### Electrophysiology

Whole-cell patch clamp electrophysiological recordings of neurons at days-in-vitro (DIV) 13-20 were performed at room temperature. For these recordings, neurons were bathed in an extracellular solution composed of 140 mM NaCl/3 mM KCl/2 mM CaCl_2_/10 mM Na-HEPES/1.0 μM tetrodotoxin (TTX; Sigma-Aldrich, St. Louis, MO)/20 mM dextrose, pH 7.35. Borosilicate glass recording pipettes (Drummond Scientific, Broomall, PA), with a resistance of 2-4 MΩ, were constructed using a Flaming/Brown Micropipette Puller, Model P-97 (Sutter Instrument Company). Pipettes were back-filled with an intracellular solution of 140 mM CsF/2 mM CaCl_2_/10 mM EGTA/10 mM HEPES/2 mM tetraethylammonium chloride/4 mM Na_2_ATP, pH 7.35. The cells were visualized using a Nikon Eclipse TE200 microscope. The test solutions were applied using a nine-barrel Rapid Solution Changer, RSC-200 (Biologic, Claix, France/Molecular Kinetics, Pullman, WA). The extracellular solution, containing 100 μM NMDA/10 μM Gly/1 μM TTX/0.5 μM strychnine, was applied to the neurons for 3 sec to elicit NMDA-induced currents. The neurons were washed free of agonists with extracellular solution and then perfused with 2 μM of ConPr1, ConPr2, or ConPr3 for 5 min, after which the NMDA-induced currents were recorded again. For the controls, similar recordings were accomplished with 5 μM ConG and scrambled peptide for 3 and 5 min, respectively. An Axopatch-200B amplifier (Axon Instruments, Foster City, CA) was used to record the whole-cell current, low-pass filtered at 5 kHz by a built-in, eight-pole Bessel filter, digitized at 1 kHz sampling frequency using a Digidata 1322A signal conditioning amplifier (Axon Instruments). Cells were voltage-clamped at -70 mV, pH 7.35. pCLAMP-8 software (Axon Instruments, Sunnyvale, CA) was used to acquire data. Data were analyzed utilizing Clampfit and Prism Graph Pad.

### Western blots

At 15 hr prior to treatment with agonists or antagonists, one-half of the medium of DIV 16 rat hippocampal neurons was exchanged with fresh Neurobasal medium without the B27 supplement and 1 μM TTX was added to the culture. The neurons were then pretreated with 40 μM CNQX and 5 μM nifedipine for 20-30 min, then exposed to 5 μM ConPr1, ConPr2, or ConPr3, for 5 min, and finally stimulated with 50 μM NMDA for 5 min. After this step, cell lysates were obtained by first washing the neurons with PBS and then lysing the cells directly with 60 μl of SDS gel loading buffer. The fractionated samples were probed for phospho (P)-CREB (Ser^133^) using rabbit-anti-P-CREB (Cell Signaling Technology, Danvers, MA) and total CREB (loading control) using rabbit-anti-CREB (48H2, Cell Signaling Technology). The bands were visualized by chemiluminescence. Densitometric analyses of P-CREB (Ser^133^) and total-CREB were performed utilizing ImageJ program 1.46r (NIH, USA http://imagej.nih.gov/ij). The intensities of the bands were read as the areas under the density curve. The values obtained were used to calculate the ratio of P-CREB (Ser^133^)/CREB.

Murine neuronal cultures from WT, GluN2A^-/-^, and GluN2B^-/-^ genotypes harvested at DIV 15-18 were lysed and subjected to Western blot analysis to probe the expression of GluN1, GluN2A, GluN2B, and GluN2D. α-tubulin levels probed separately served as the loading controls. The 1° antibodies used were rabbit-anti-GluN1 (Cell Signaling Technology), rabbit-anti-GluN2B (Neuromab Antibodies, UC-Davis, CA), mouse-anti-GluN2A (BD Transduction, San Jose, CA), rabbit-anti-GluN2D (Sigma-Aldrich), and mouse-anti-α-tubulin (Santa Cruz Biotechnology, Santa Cruz, CA). Corresponding HRP-conjugated 2° antibodies used were rabbit-anti-IgG (Cell Signaling Technology), mouse-anti-IgG, and mouse-anti-IgM (Santa Cruz Biotechnology). Western blots were developed using Clarity^TM^ Western ECL substrate (Bio-Rad, Hercules, CA) and the images were acquired using ChemiDoc^TM^ MP Imaging system from (Bio-Rad). The intensities of GluN2D and tubulin bands were measured as volume intensity using the Auto-Analysis tool of the Image Lab 4.1 software (Bio-Rad). The values obtained were used to calculate the ratio of GluN2D/tubulin. 

### Statistical analyses

All experiments were replicated at least 3X with independent samples unless otherwise specified and data are expressed as mean values ± S.E.M. Statistical analysis was performed using a two-tailed unpaired Student’s t test with Excel software. Differences were considered significant at *p* < 0.05.

## Results

### Structural characterization of ConPr1, ConPr2, and ConPr3

**Figure 1:**
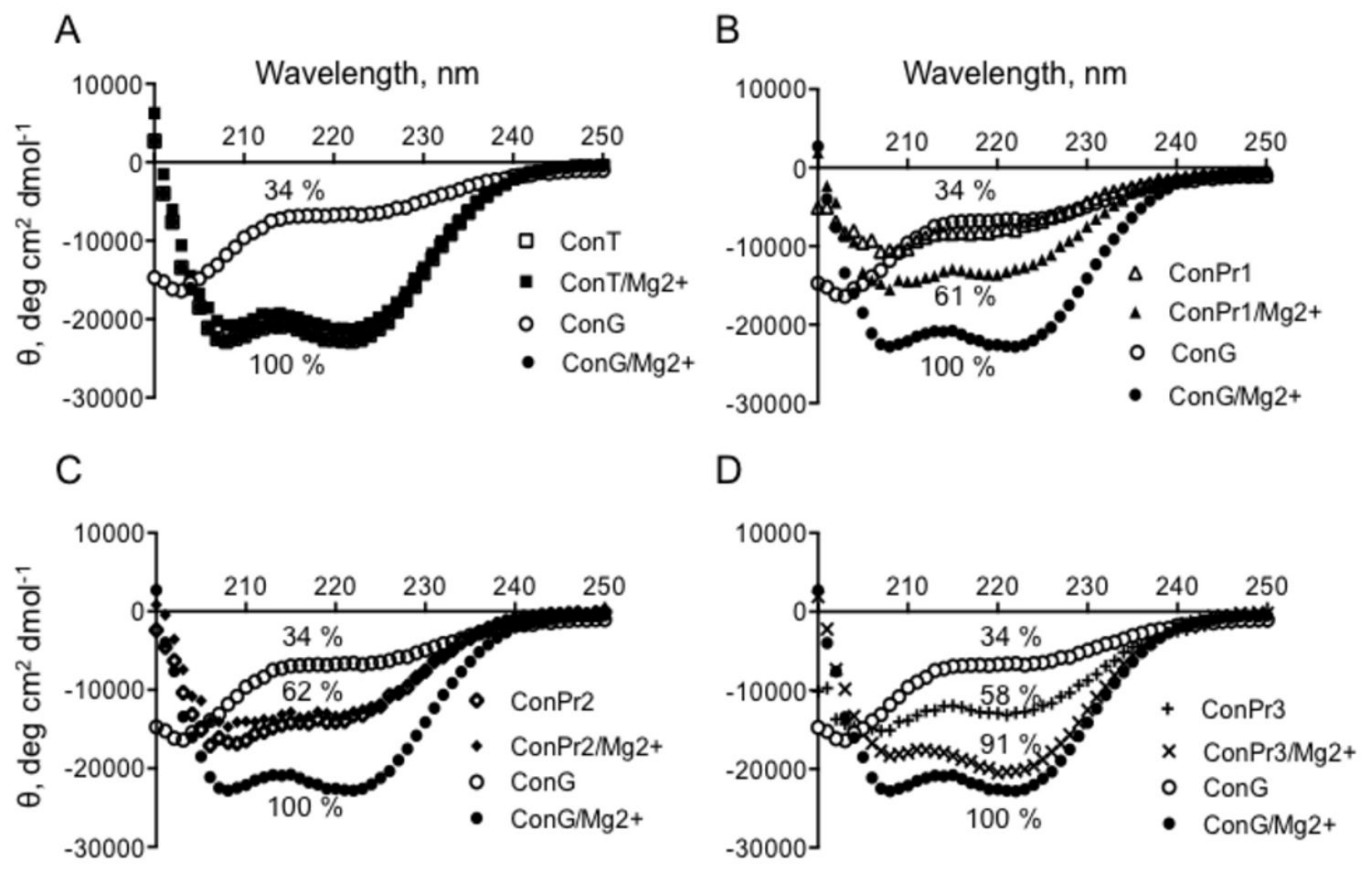
Representative CD spectra of *C*. *parius*-derived conantokins. Scans from 200-250 nm of: (A) ConT or ConG ± MgCl_2_; (B) ConG or ConPr1 ± MgCl_2_; (C) ConG or ConPr2 ± MgCl_2_; (D) ConG or ConPr3 ± MgCl_2_. The peptide concentrations were 90 μM and the MgCl_2_ concentration was 2 mM, when present. The buffer was 10 mM HEPES, pH 7, at 25° C. Each curve represents an average of three scans and the percent helicity is determined as a percent of molar ellipticity of -22,081 deg cm^2^ dmol^-1^, which is observed for ConG + 2 mM MgCl_2_ and which represented 100% α-helicity of these classes of peptides.

Conantokins, *via* their highly conserved Gla residues, interact with divalent ions [28], such as Mg^2+^ and Ca^2+^. These interactions enable the peptides to adopt α-helical conformations [29]. The *C. parius*-derived conantokins synthesized in our laboratory were characterized qualitatively for α-helicity by CD spectroscopy, in the absence and presence of Mg^2+^, and compared to published data [22,28,30,31]. The data revealed that ConPr1 and ConPr3 showed a Mg^2+^-dependent increase of α-helical character, as reflected by the negative increase in the molar ellipticity at 222 nm, to an extent trending toward that of Mg^2+^/ConG and apo- and divalent cation-loaded ConT (Figure 1A), each of which is known to exist as an end-to-end α-helix [5,29,30,32,33]. Since CD analytical algorithms would not likely be reliable for ~20 residue peptides, the α-helicity changes of ConPr1, ConPr2, and ConPr3, as a result of divalent cation binding, were calculated by taking the θ_222 nm_ of apo-ConT and Mg^2+^-loaded ConG as reflecting 100% helicity, and calculating the relative amount of α-helix in the *C. parius* peptides by the ratio of their observed θ_222 nm_ values to that of Mg^2+^/ConG. The values obtained are shown in the labeled curves of Figure 1B-D. From the data obtained, we conclude that modest increases in α-helicity are observed for ConPr1 and ConPr3, in the presence of Mg^2+^, with ConPr3 (Figure 1D) displaying the highest relative α-helical content of the *C. parius* conantokins in the presence of Mg^2+^. ConPr2 did not undergo a measureable alteration upon addition of Mg^2+^. 

### Inhibition of NMDA induced currents by C. parius conantokins on rat hippocampal and mouse cortical neurons

**Figure 2:**
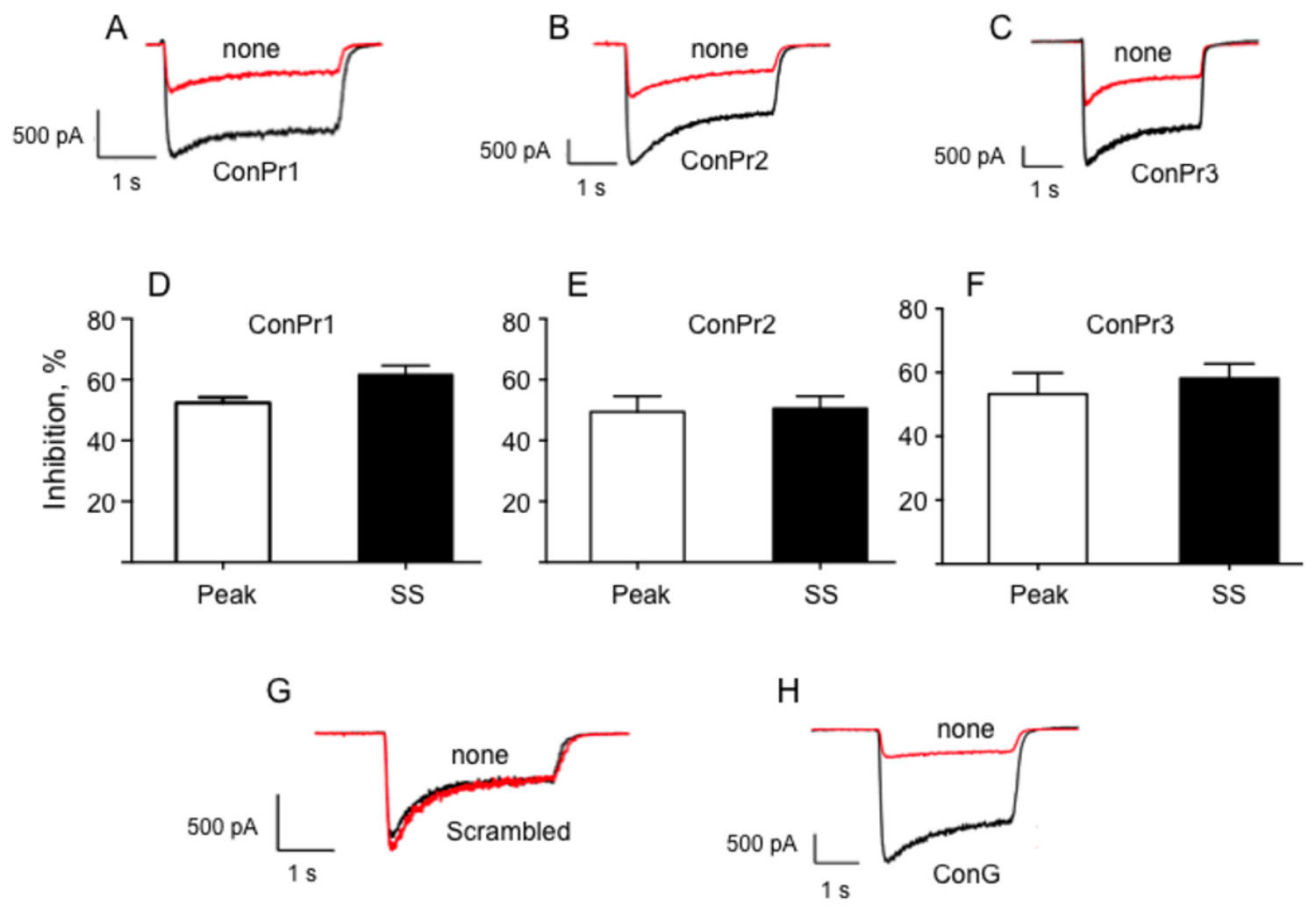
Inhibition of the NMDA/Gly-induced current by *C*. *parius*-derived conantokins in rat hippocampal neurons. Representative traces of the NMDA-induced currents using DIV 13-20 primary rat hippocampal neurons in the presence or absence of *C. parius* conantokins. (A) ConPr1. (B) ConPr2. (C) ConPr3. In each case, 2 μM solutions of antagonist peptides were perfused for 5 min. NMDAR currents were recorded before (black) and after (red) the conantokin perfusions. (D-F) The bar graphs represent the percentage inhibition observed for both peak and steady state (SS) components of NMDAR current for *C. parius* conantokins, (D) ConPr1, (E) ConPr2, (F) ConPr3. For control experiments, (G) 5 μM scrambled peptide (based on the composition of ConPr1) was perfused for 5 min, or (H) 5 μM ConG was perfused for 3 min. The data are averages of N = 7 separate neurons for ConPr1, N = 10 for ConPr2, N = 10 for ConPr3, and N = 3 for ConG and for the scrambled peptide.

The physiological role of NMDARs in the hippocampus and neocortex has been widely studied regarding the link between synaptic transmission and plasticity that these receptors elicit post-activation [34]. In order to evaluate the antagonistic effects of these conantokin peptides on NMDARs, and accurately demonstrate *in vivo* cellular responses, we utilized DIV 13-20 rat hippocampal and mouse cortical neurons. DIV 13-20 neurons are routinely utilized for studying NMDAR-mediated potentiation or signaling, since they express functional NMDARs. Previously, age-dependent sensitivity of NMDAR-mediated whole cell currents to subunit (GluN2B)-selective ifenprodil and ConG, and GluN2A-selective NVP-AAM077, have been utilized to characterize NMDAR subunits for DIV 12-19 neurons [23,35,36]. Simultaneous perfusion of ConPr peptides, along with NMDA, resulted in a slow onset rate of inhibition of the NMDA-induced currents. In order to avoid a longer period of NMDA stimulation, and any associated effects on the patched neuron, the NMDA responses were recorded before and after inhibitor perfusion. Rat hippocampal neurons were stimulated with NMDA/Gly for 3 sec. Significant inhibition was observed in both peak and steady-state currents when agonist stimulation was recorded after the neurons were perfused with ConPr1, ConPr2, and ConPr3 for 5 min (Figure 2A-F). Interestingly, a slightly slower desensitization time constant of the NMDARs was observed in the presence of conantokins (data not shown). To show the specificity of the *Conus* peptides towards inhibiting NMDAR-mediated currents, experiments were performed with a scrambled peptide of the same amino acid composition as ConPr1, which did not inhibit NMDA-evoked currents in rat hippocampal neurons (Figure 2G). The well-studied ConG, that robustly inhibits NMDAR-mediated currents, was used as a control peptide (Figure 2H).

**Figure 3:**
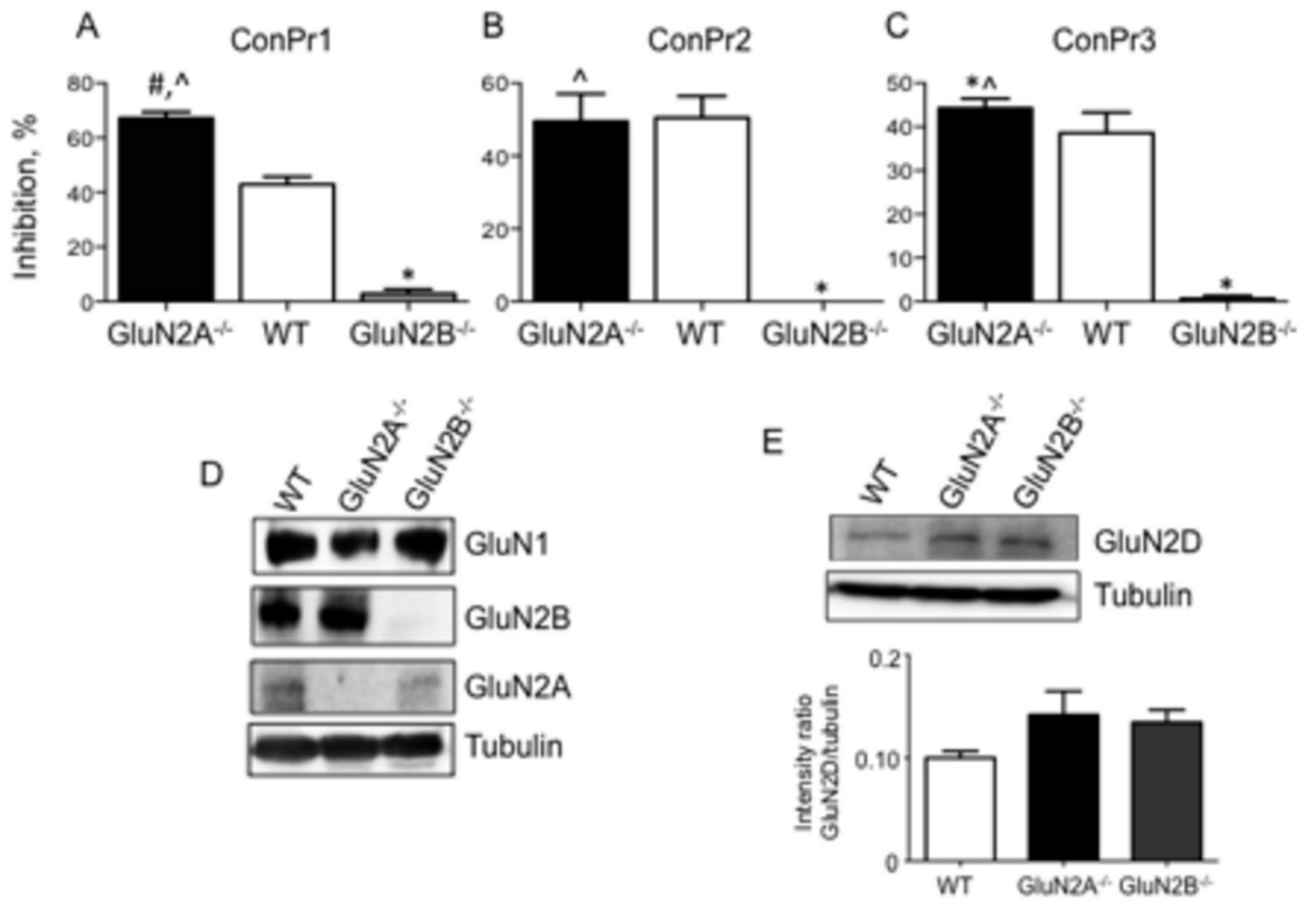
Lack of inhibition of NMDA-induced currents by *C*. *parius*-derived peptides in GluN2B^-/-^ mouse cortical neurons. DIV 13-20 primary cortical neurons from WT, GluN2A^-/-^, and GluN2B^-/-^ mouse brains were utilized to observe inhibition of NMDA-induced currents. (A) ConPr1. (B) ConPr2. (C) ConPr3. The percent inhibition of NMDAR currents in the absence of conantokins is plotted. The antagonist conantokins (2 μM) were perfused for 5 min and NMDAR currents were recorded before and after the perfusion. Data were obtained from at least three independent recordings for each genotype. (D) Western blot of neuronal cell lysates probed with anti-GluN1, anti-GluN2B, or anti-GluN2A. The lysates were also probed for α-tubulin, shown in lower panel, as loading controls. (E) Representative Western blot and densitometric analysis of WT, GluN2A^-/-^, and GluN2B^-/-^ cells probed with anti-GluN2D. α-tubulin is the loading control. The bar graph display the means ± SEM of 3 independent experiments. *p < 0.05 for comparison between GluN2B^-/-^ and WT neurons, #p < 0.05 between GluN2A^-/-^ and WT neurons, *^p* < 0.05 between GluN2A^-/-^ and GluN2B^-/-^ neurons.

 The NMDAR is a heterotetrameric complex of NR1 and NR2 subunits [37]. Previous studies utilizing a heterologous NMDAR expression system in *Xenopus* oocytes have demonstrated that ConPr1, ConPr2, and ConPr3 inhibited Glu/Gly-evoked currents to the highest degree for receptors containing the GluN2B subunit in these cells [22]. Since this system is unrelated to neuronal cells with regard to interdependence of NMDAR subunit expression, we further examined the effects of an NMDAR subunit gene deletion on inhibition by the conantokins through comparison of the amplitudes of excitatory currents in cortical neurons from GluN2A^-/-^ and GluN2B^-/-^ mice. The currents measured after perfusion with 2 μM ConPr1, ConPr2, or ConPr3 for 5 min are reported as % inhibition of NMDA-mediated currents (Figure 3A-C). Total ablation of the GluN2B subunit resulted in almost no inhibition by any of the tested *C. parius* conantokins, indicating that the inhibitory activity of these peptides in brain cells is chiefly mediated by the GluN2B subunit in neurons. Although no significant differences in the inhibitory properties of ConPr2 or ConPr3 were observed between WT and GluN2A^-/-^ neurons (both ~40-50%), inhibition by ConPr1 was significantly more robust in GluN2A^-/-^ neurons (~70%), compared to WT neurons (~40%). This result cannot be explained by the higher levels of GluN2B subunit in age-matched GluN2A^-/-^ neurons, compared to WT neurons, since neurons from both the genotypes showed comparable levels of GluN2B and GluN1 subunits (Figure 3D). It has been shown that ConPr1 potently inhibited GluN2D-containing receptors in the heterologous *Xenopus* oocytes [22]. Thus, we compared the levels of GluN2D subunit in mature (DIV 15) WT, GluN2A^-/-^, and GluN2B^-/-^ neurons by immunoblot analyses. These NMDAR subunits were found to be present at similar levels in each of the cell lines (Figure 3E). Thus, the selective potency of ConPr1 for increased inhibition of GluN2A^-/-^ neurons cannot be explained by differences in either GluN2B or GluN2D levels. In this regard, it is possible that these peptides could demonstrate differential inhibitory properties towards the GluN2D subunit in a heterologous system that solely express the transfected receptor subunits, compared to neurons, which express a dynamic range of different subunit combinations. However, our data regarding GluN2B-selectivity of ConPr1, ConPr2, and ConPr3 in neurons are consistent with published findings with recombinant receptors in cells [22], an important advance in knowledge in a more pathophysiological setting.

### The effect of C. *parius*-derived conantokins on iCa^2+^ levels in hippocampal and cortical neurons

**Figure 4:**
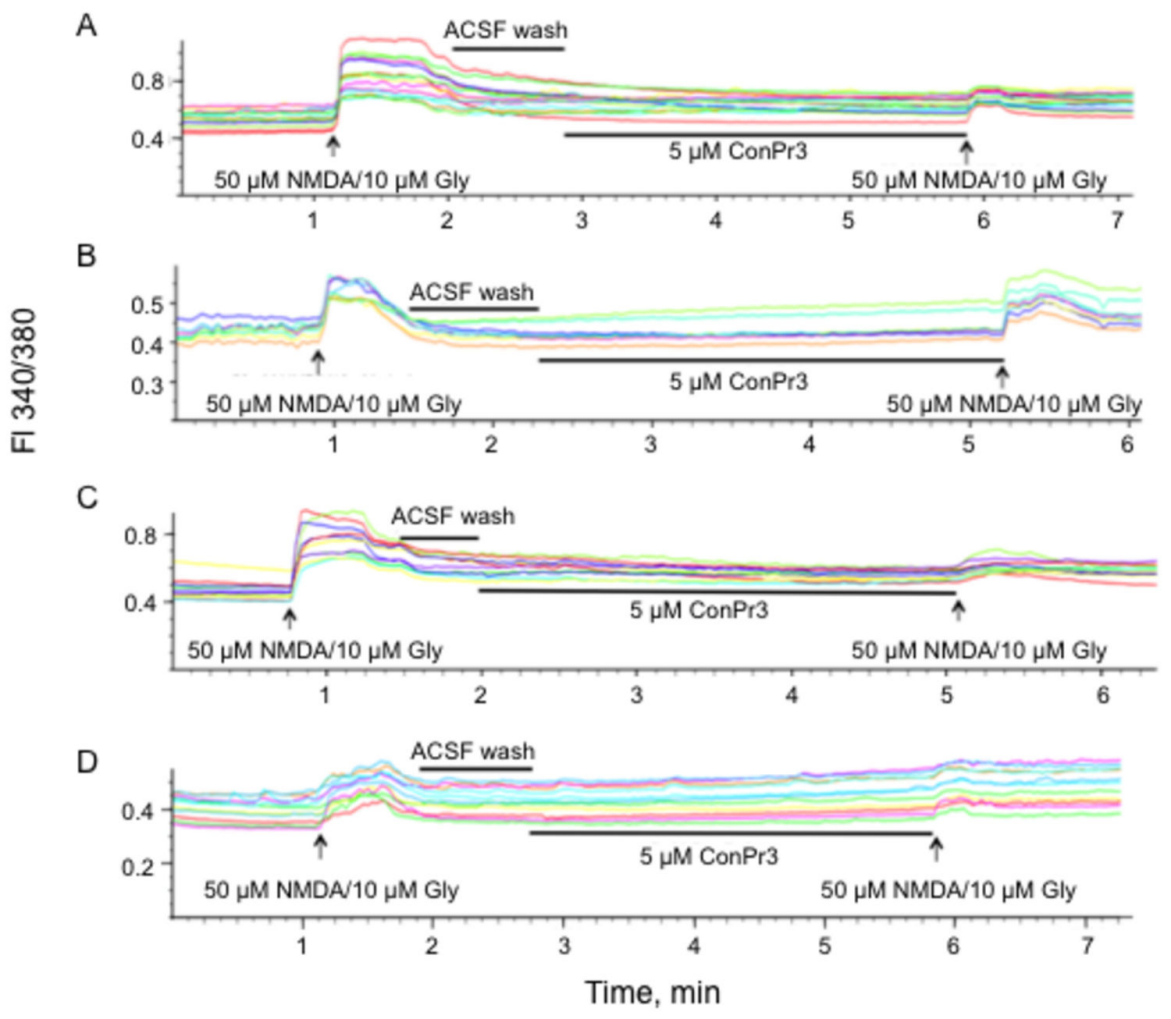
Representative traces of the effect of ConPr3 on NMDA/Gly-dependent Ca^2+^ influx in neurons. (A) DIV 12-15 rat hippocampal neurons. (B) WT mouse cortical neurons. (C) GluN2A^-/-^ mouse cortical neurons. (D) GluN2B^-/-^ mouse cortical neurons. The cells were first stimulated with 50 μM NMDA/10 μM glycine, then freed of coagonists by washing with ACSF buffer. Lastly, the neurons were treated with 5 μM ConPr3 for 3 min. The neurons were then again stimulated with 50 μM NMDA/10 μM Gly to determine the extent of inhibition of Ca^2+^ influx. Changes in iCa^2+^ were monitored by changes in the fluorescence (FI) ratio at 340/380 nm.

**Figure 5:**
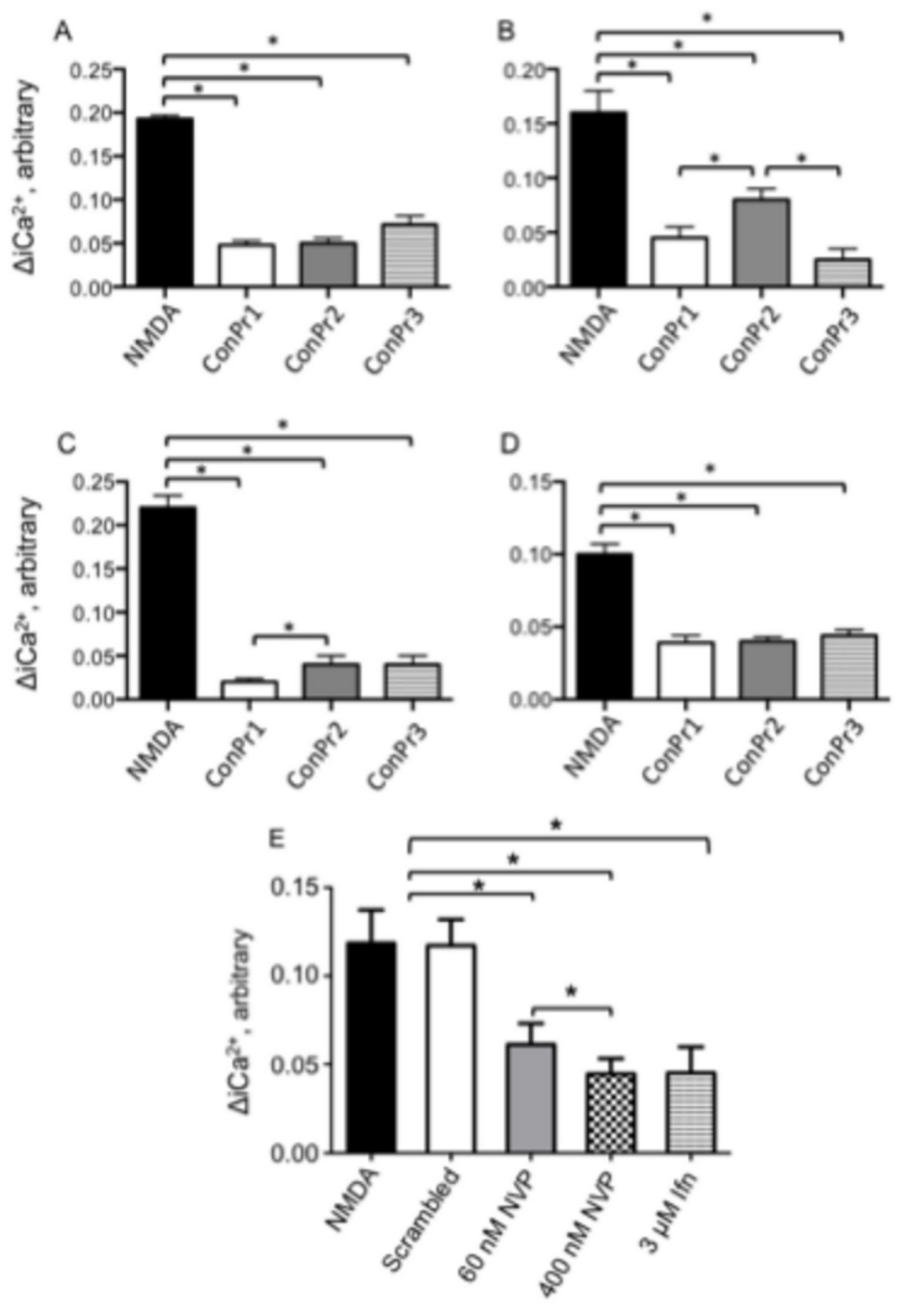
ConPr1 inhibited Ca^2+^ influx into cultured neurons most robustly in GluN2A^-/-^ cortical neurons. (A) Inhibition of NMDA/Gly-dependent Ca^2+^ influx in DIV 12-15 rat hippocampal neurons. (B) DIV 12-15 WT mouse cortical neurons. (C) DIV 12-15 mouse GluN2A^-/-^ cortical neurons. (D) DIV 12-15 mouse GluN2B^-/-^ cortical neurons, after exposure to 5 μM ConPr1, ConPr2, or ConPr3 for 3 min and then re-stimulation with 50 μM NMDA/10 μM glycine. (E) Effect of 5 μM scrambled peptide (N = 8), 60 nM NVP-AAM077 (NVP) (N = 15), 400 nM NVP-AAM077 (NVP) (N = 15), and 3 μM ifenprodil (N = 8) on NMDA/Gly-dependent Ca^2+^ influx in DIV 12-15 rat hippocampal neurons. The bar graphs display mean ± S.E.M of 2-3 independent experiments performed using different batches of neurons and one batch of neurons (N = 8) for scrambled peptide. *p < 0.05 for comparison between NMDA treated neurons and neurons pre-incubated with ConPr1, ConPr2, and ConPr3. For panel E, no significant differences were observed in [iCa^2+^] changes between neurons treated with NMDA alone and with neurons treated with NMDA/scrambled peptide. In panel E, *p < 0.05 for pairwise comparisons between NMDA alone or NMDA/scrambled peptide and neurons treated with 60 nM NVP-AAM077, 400 nM NVP-AAM077, 3 μM ifenprodil.

The NMDAR is relieved of its Mg^2+^ block during membrane depolarization as a consequence of Glu/Gly co-agonism [38]. This results in Ca^2+^ influx through activated NMDAR channels that can serve as either a signal for promoting survival or for promoting cell death [39,40]. In light of the importance of blockage of the NMDAR under pathological conditions, the effects of ConPr1, ConPr2, and ConPr3 were tested on NMDA/Gly-mediated Ca^2+^ influx into mature neurons. A robust increase in iCa^2+^ was observed upon stimulation of DIV 13-15 primary rat hippocampal neurons with 50 μM NMDA/10 μM Gly (Figure 4A). *In vivo* biological activity studies performed in mice suggests slower inhibitory properties for *C. parius*-conantokins in comparison to ConG [22]. Our initial control experiments were carried out using varying concentrations (5 μM, 10 μM, and 20 μM) of the *Conus* peptides, with pre-incubation times of either 1 or 3 min. It was observed that pre-incubation of 5 μM peptide for 3 min prior to a second application of co-agonist solution showed optimum inhibition without saturation. Representative traces, shown only for ConPr3, demonstrated that iCa^2+^ influx was significantly reduced after treatment with the antagonist (Figures 4A, 5A). Similar data were obtained with ConPr1 and ConPr2 (Figure 5A). The presence of the scrambled analog of ConPr1 (5 μM) did not result in any decrease in iCa^2+^ levels (Figure 5E), thus further demonstrating the specific inhibition of the NMDARs by these *C. parius* peptides. Additional controls that were included to study inhibition of iCa^2+^ influx consisted of the known GluN2A-specific antagonist NVP-AAM007 (at low concentrations) and GluN2B-specific antagonist, ifenprodil in rat hippocampal neurons. Compared to iCa^2+^ inhibition by ConPr1 (75%), ConPr2 (75%), and ConPr3 (63%); inhibition by 60 nM NVP-AAM007 that specifically inhibits the GluN2A subunit [41], was 46%. When 400 nM of NVP-AAM007 was used, which renders this agent non-subunit specific [41], inhibition of iCa^2+^ influx was increased (64%) to almost that observed by the *C. parius* peptides. Similar levels (64%) of inhibition of iCa^2+^ influx were observed by 3 μM ifenprodil (Figure 5E). These data suggest that robust inhibition of iCa^2+^ influx is best mediated by antagonism of the GluN2B subunit. To further determine the GluN2-subunit selectivity of these conantokins, iCa^2+^ levels were measured in DIV 12-15 GluN2A^-/-^ and GluN2B^-/-^ mouse cortical neurons, and compared to DIV 12-15 WT neurons (Figure 4B-D). It was observed that GluN2A^-/-^ neurons showed maximum inhibition of iCa^2+^ influx after treatment with 5 μM ConPr1 (91%), ConPr2 (82%), and ConPr3 (82%) for 3 min, when compared to WT neurons [ConPr1 (72%), ConPr2 (50%), ConPr3 (85%)], or GluN2B^-/-^ neurons [ConPr1 (61%), ConPr2 (60%), ConPr3 (56%)] (Figure 5B-D). From these data, we conclude that inhibition of iCa^2+^ influx by the *C. parius* peptides was not as robust in GluN2B^-/-^ neurons as in GluN2A^-/-^ neurons, indicating the GluN2B-specificity of this class of peptides.

### Effects of conantokins on CREB phosphorylation

**Figure 6:**
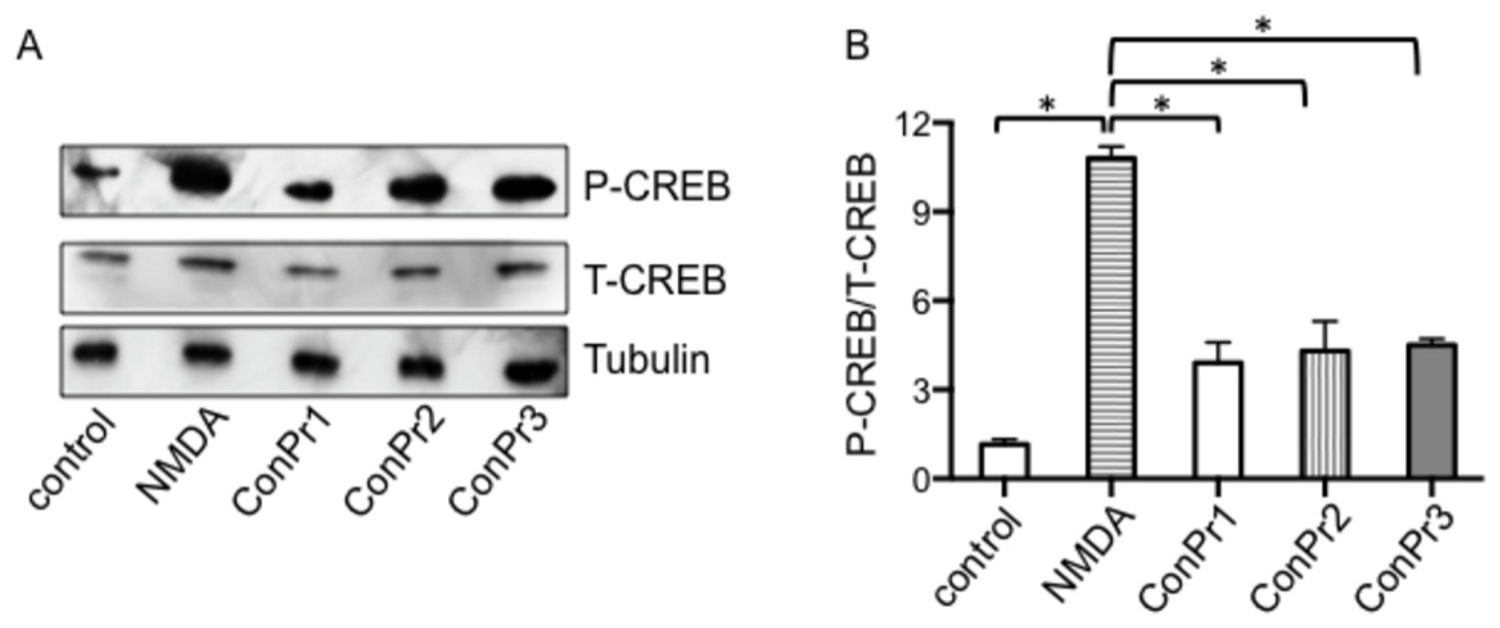
*Conus* *parius*-derived conantokins diminished NMDA/Gly-stimulated P-CREB levels in neurons. (A) Representative Western blot of cultured rat hippocampal neurons at DIV 16 that were not stimulated (control), stimulated with 50 μM NMDA, or pre-incubated with 5 μM ConPr1, ConPr2, or ConPr3 for 5 min, and then stimulated with 50 μM NMDA for 5 min. Total cell lysates were obtained and immunoblotted for P-CREB and total CREB (T-CREB). α-tubulin was probed as loading control. (B) Densitometric analysis of the blots representing the ratios of P-CREB/T-CREB in the samples.

A change in iCa^2+^ *via* influx through NMDAR channels modifies a number of downstream signaling events that are involved in synaptic plasticity and survival [39,40,42]. In particular, phosphorylation of CREB protein (P-CREB) at Ser^133^ is central in formation of long-term synapses and transcription of genes that promote neuron survival [43,44]. We previously reported that ConG, ConT, and ConR affects NMDA/Gly-mediated phosphorylation of CREB of neurons in a development-dependent manner [23]. However, for the current study, mature rat hippocampal neurons at DIV 16 were utilized to evaluate the effects of *C. parius* peptides on CREB phosphorylation. Neurons at DIV 16 were treated with 20 μM of each conantokin for 5 min, stimulated with NMDA/Gly, and then immunoblotted for P-CREB(Ser^133^) levels. It was observed that these conantokins inhibited phosphorylation of CREB at Ser^133^ by 64% (ConPr1), 61% (ConPr2), and 59% (ConPr3) (Figure 6A,B). This indicates that while the antagonist function of these peptides on the NMDAR diminishes iCa^2+^ influx, they do not completely eliminate the CREB signaling pathway that is essential for neuronal maintenance. 

## Discussion and Conclusions

To date, conantokins from a variety of cone snail species have been studied with respect to their structural and inhibitory properties of neuronal ion channels. Special emphasis has been placed on understanding of the differential selectivity towards specific GluN subunits, especially GluN2B, and subsequent downstream signaling events. In this study, we have utilized neurons from rat hippocampus, and cortical neurons from WT, GluN2A^-/-^, and GluN2B^-/-^ mice to evaluate inhibitory and signaling properties of the *C. parius* peptides. 

 Conantokins, with an optimum distribution of Gla residues, facilitate their binding to divalent ions, resulting in α-helical structures capable of dimerization. The lack of a defined structure in apo-ConG, and similar peptides, has been attributed to i, i+4, i+7, and/or i+11 spacing of Gla residues, with consequent charge repulsion [5]. However, this same spacing also allows for divalent cation binding and consequent adoption of an end-to-end helical peptide [31-33]. The percent helicity values for the *C. parius* family of conantokins have been reported relative to θ_222_ value of Mg^2+^/ConG, taken as a standard for 100% helix in these closely related peptides. This comparison to well-established structures of ConG, that represents full (Mg^2+^/ConG) or no (apo-ConG) α-helix values, enabled a relative quantitative assessment of the CD data. ConPr3, like ConT, contains a Lys at position 7, thus neutralizing the charge repulsion with Gla^4^, when Gla^7^ is present, as in ConG. This feature allows apo-ConPr3 to adopt a partial helical conformation, similarly to the case to ConT [6]. *C. parius* peptides also differ from ConG in the absence of a Gla residue at amino acid sequence position-3, with ConPr1 displaying an Asp residue at that location, and ConPr2 and ConPr3 containing a 4-trans-hydroxyproline residue at sequence position-3. The Gla^3^ in ConG also contributes to charge repulsion and opening of its apo-conformation, and likely explains its comparatively low α-helicity found in the metal-free form. The physiological or pharmacological role of divalent cations and the α-helical conformation of the conantokin class of peptides are still ambiguous. Earlier research has shown, that both ConG and ConR required divalent cation binding to promote stability of the α-helical structure, whereas, ConT is able to form a stable α-helical structure in the presence or absence of divalent cations [7,33]. It has been suggested that while in peptides, like ConT, divalent cation binding may not be required for structural stability, divalent cations may bind to the Gla side-chains contributing towards the neuroactivities of these peptides in combination with receptors [7]. 

 The differential NMDAR subunit selectivity of the conantokins reported herein have been determined from patch clamp experiments on *Xenopus* oocytes or HEK293 cells co-expressing GluN1 and the GluN2 subunits of interest [22,45-47], cells that are not native to the receptors. This could certainly affect the properties of NMDAR subunits in such non-natural environments. Our laboratory has recently reported the inhibitory effects of ConG, ConT, and ConR on developing rat hippocampal neurons [23]. In the current study, we have utilized rat hippocampal neurons and mouse cortical neurons for electrophysiology and ratiometric Ca^2+^ imaging experiments to further define the antagonistic properties of ConPr1, ConPr2, and ConPr3. The NMDA/Gly-stimulated current in the presence of *C. parius* conantokins directly correlates with inhibition of NMDA/Gly-mediated Ca^2+^ influx through NMDAR channels, whereas control experiments with scrambled peptide derived from ConPr1 shows no such inhibitory activity (Figures 2I, 5E). Utilization of GluN2A^-/-^ and GluN2B^-/-^ neurons demonstrated that the GluN2B subunit is critical for robust inhibition by the *C. parius* peptides in neurons. These findings suggest that GluN2B-mediated neuronal excitoxicity can be potentially mitigated by GluN2B-specific antagonists [42,47,48]. Total abrogation of the GluN2A subunit did not significantly affect the NMDA/Gly-induced current inhibition by ConPr2 or ConPr3, when compared to WT neurons. However, ConPr1 displayed increased inhibition of NMDAR current in the GluN2A^-/-^ neurons. Other structural features, such as the presence of Y^5^ in ConPr1 that increases its inhibitory potency towards GluN2B or GluN2D subunits, have previously been considered [22]. Thus, it is consistent that in the absence of the GluN2A subunit, ConPr1 demonstrated ~30% more potency towards the GluN2B or GluN2D subunits in the GluN2A^-/-^ neurons. 

The role of transcription factor CREB in neuronal survival, neurogenesis, and synaptic plasticity is well documented [49]. Although, the *C. parius* peptides decreased iCa^2+^ levels by ~62% (average of inhibition of iCa^2+^ influx levels of ConPr1, -2, and -3 in rat hippocampal neurons), compared to ConG/T/R in age-matched neurons [23], the levels were sufficiently stimulated to act as a secondary messenger and allow phosphorylation of CREB. This suggests that the mechanism of action of the *C. parius* peptides from ConG/T/R may differ at the molecular level. Nonetheless, it is of significance to note that the antagonist function of ConPr1, ConPr2, or ConPr3 can sustain the Ca^2+^/P-CREB integration axis that is critical for regulating genes containing CRE elements and eventually neuronal survival [50]. 

 In conclusion, our data have generated insights in natural cells regarding the mechanisms of the inhibitory properties of the *C. parius* conantokins that can serve as potential drug candidates against an overactive NMDAR in various neuropathologies. The differential selectivity for the GluN2B subunit by the *C. parius* peptides, along with ConG and ConRl-B [51], can be further explored, pharmacologically, in comparison to subunit-non-specific conantokins. Toward this end, a stapled (dicarba-bridged) ConG, that showed 100-fold greater potency for the GluN2B subunit and had potent anti-convulsant properties, has been synthesized and characterized [52]. Thus, biophysical and cellular characterization studies enable correlations of the structure-function relationships of conantokins, which may have applicability as pharmacological allosteric NMDAR ion channel blockers. Future directions include evaluating the neuroprotective potential of these set of conantokins, *in vivo* using stroke models in rodents.

## References

[1] Terlau H, Olivera BM (2004) Conus venoms: a rich source of novel ion channel-targeted peptides. Physiol Rev 84: 41-68. doi:10.1152/physrev.00020.2003. PubMed: 14715910. 14715910

[2] McIntosh JM, Olivera BM, Cruz LJ, Gray WR (1984) γ-Carboxyglutamate in a neuroactive toxin. J Biol Chem 259: 14343-14346. PubMed: 6501296. 6501296

[3] Haack JA, Rivier J, Parks TN, Mena EE, Cruz LJ et al. (1990) Conantokin-T. A gamma-carboxyglutamate containing peptide with N-methyl-d-aspartate antagonist activity. J Biol Chem 265: 6025-6029. PubMed: 2180939. 2180939

[4] White HS, McCabe RY, Armstrong H, Donevan SD, Cruz LJ et al. (2000) In vitro and in vivo characterization of conantokin-R, a selective NMDA receptor antagonist isolated from the venom of the fish-hunting snail Conus radiatus. J Pharmacol Exp Ther 292: 425-432. PubMed: 10604979. 10604979

[5] Dai Q, Sheng Z, Geiger JH, Castellino FJ, Prorok M (2007) Helix-helix interactions between homo- and heterodimeric gamma-carboxyglutamate-containing conantokin peptides and their derivatives. J Biol Chem 282: 12641-12649. doi:10.1074/jbc.M609087200. PubMed: 17347154. 17347154

[6] Prorok M, Warder SE, Blandl T, Castellino FJ (1996) Calcium binding properties of synthetic gamma-carboxyglutamic acid-containing marine cone snail "sleeper" peptides, conantokin-G and conantokin-T. Biochemistry 35: 16528-16534. doi:10.1021/bi9621122. PubMed: 8987986. 8987986

[7] Skjaerbaek N, Nielsen KJ, Lewis RJ, Alewood P, Craik DJ (1997) Determination of the solution structures of conantokin-G and conantokin-T by CD and NMR spectroscopy. J Biol Chem 272: 2291-2299. doi:10.1074/jbc.272.4.2291. PubMed: 8999936. 8999936

[8] Prorok M, Geng JP, Warder SE, Castellino FJ (1996) The entire gamma-carboxyglutamic acid- and helical stack-domains of human coagulation factor IX are required for optimal binding to its endothelial cell receptor. Int J Pept Protein Res 48: 281-285. PubMed: 8897096. 8897096 10.1111/j.1399-3011.1996.tb00842.x

[9] Cull-Candy S, Brickley S, Farrant M (2001) NMDA receptor subunits: diversity, development and disease. Curr Opin Neurobiol 11: 327-335. doi:10.1016/S0959-4388(00)00215-4. PubMed: 11399431. 11399431

[10] Chatterton JE, Awobuluyi M, Premkumar LS, Takahashi H, Talantova M et al. (2002) Excitatory glycine receptors containing the NR3 family of NMDA receptor subunits. Nature 415: 793-798. doi:10.1038/nature715. PubMed: 11823786. 11823786

[11] Cull-Candy SG, Leszkiewicz DN (2004) Role of distinct NMDA receptor subtypes at central synapses. Science STKE 2004: re16. 10.1126/stke.2552004re16 15494561

[12] Lipton SA (1994) Neuronal injury associated with HIV-1 and potential treatment with calcium-channel and NMDA antagonists. Dev Neurosci 16: 145-151. doi:10.1159/000112101. PubMed: 7705221. 7705221

[13] Arundine M, Aarts M, Lau A, Tymianski M (2004) Vulnerability of central neurons to secondary insults after in vitro mechanical stretch. J Neurosci 24: 8106-8123. doi:10.1523/JNEUROSCI.1362-04.2004. PubMed: 15371512. 15371512 PMC6729801

[14] Arzberger T, Krampfl K, Leimgruber S, Weindl A (1997) Changes of NMDA receptor subunit (NR1, NR2B) and glutamate transporter (GLT1) mRNA expression in Huntington's disease--an in situ hybridization study. J Neuropathol Exp Neurol 56: 440-454. doi:10.1097/00005072-199704000-00013. PubMed: 9100675. 9100675

[15] Möddel G, Jacobson B, Ying Z, Janigro D, Bingaman W et al. (2005) The NMDA receptor NR2B subunit contributes to epileptogenesis in human cortical dysplasia. Brain Res 1046: 10-23. doi:10.1016/j.brainres.2005.03.042. PubMed: 15890316. 15890316

[16] Wei J, Dong M, Xiao C, Jiang F, Castellino FJ et al. (2006) Conantokins and variants derived from cone snail venom inhibit naloxone-induced withdrawal jumping in morphine-dependent mice. Neurosci Lett 405: 137-141. doi:10.1016/j.neulet.2006.06.040. PubMed: 16859831. 16859831

[17] Kemp JA, McKernan RM (2002) NMDA receptor pathways as drug targets. Nat Neurosci 5 Suppl: 1039-1042. doi:10.1038/nn936. PubMed: 12403981. 12403981

[18] Malmberg AB, Gilbert H, McCabe RT, Basbaum AI (2003) Powerful antinociceptive effects of the cone snail venom-derived subtype-selective NMDA receptor antagonists conantokins G and T. Pain 101: 109-116. doi:10.1016/S0304-3959(02)00303-2. PubMed: 12507705. 12507705

[19] Xiao C, Huang Y, Dong M, Hu J, Hou S et al. (2008) NR2B-selective conantokin peptide inhibitors of the NMDA receptor display enhanced antinociceptive properties compared to non-selective conantokins. Neuropeptides 42: 601-609. doi:10.1016/j.npep.2008.09.003. PubMed: 18992939. 18992939 PMC2621068

[20] Williams AJ, Dave JR, Phillips JB, Lin Y, McCabe RT et al. (2000) Neuroprotective efficacy and therapeutic window of the high-affinity N-methyl-D-aspartate antagonist conantokin-G: In vitro (Primary cerebellar neurons) and In vivo (Rat model of transient focal brain ischemia). Studies - J Pharmacol Exp Ther 294: 378-386. 10871336

[21] Williams AJ, Ling G, Berti R, Moffett JR, Yao C et al. (2003) Treatment with the snail peptide CGX-1007 reduces DNA damage and alters gene expression of c-fos and bcl-2 following focal ischemic brain injury in rats. Exp Brain Res 153: 16-26. doi:10.1007/s00221-003-1566-6. PubMed: 12955387. 12955387

[22] Teichert RW, Jimenez EC, Twede V, Watkins M, Hollmann M et al. (2007) Novel conantokins from conus parius venom are specific antagonists of NMDA receptors. J Biol Chem 282: 36905-36913. doi:10.1074/jbc.M706611200. PubMed: 17962189. 17962189

[23] Huang L, Balsara RD, Sheng Z, Castellino FJ (2010) Conantokins inhibit NMDAR-dependent calcium influx in developing rat hippocampal neurons in primary culture with resulting effects on CREB phosphorylation. Mol Cell Neurosci 45: 163-172. doi:10.1016/j.mcn.2010.06.007. PubMed: 20600930. 20600930 PMC2923249

[24] Sakimura K, Kutsuwada T, Ito I, Manabe T, Takayama C et al. (1995) Reduced hippocampal LTP and spatial learning in mice lacking NMDA receptor epsilon 1 subunit. Nature 373: 151-155. doi:10.1038/373151a0. PubMed: 7816096. 7816096

[25] Kutsuwada T, Sakimura K, Manabe T, Takayama C, Katakura N et al. (1996) Impairment of suckling response, trigeminal neuronal pattern formation, and hippocampal LTD in NMDA receptor epsilon 2 subunit mutant mice. Neuron 16: 333-344. doi:10.1016/S0896-6273(00)80051-3. PubMed: 8789948. 8789948

[26] Colpitts TL, Castellino FJ (1994) Calcium and phospholipid binding properties of synthetic gamma-carboxyglutamic acid-containing peptides with sequence counterparts in human protein C. Biochemistry 33: 3501-3508. doi:10.1021/bi00178a006. PubMed: 8142347. 8142347

[27] Banker GA, Cowan WM (1977) Rat hippocampal neurons in dispersed cell culture. Brain Res 126: 397-425. doi:10.1016/0006-8993(77)90594-7. PubMed: 861729. 861729

[28] Blandl T, Zajicek J, Prorok M, Castellino FJ (1997) Metal ion binding properties of synthetic conantokin-G. Biochem J 328: 777-783. PubMed: 9396720. 9396720 10.1042/bj3280777 PMC1218986

[29] Prorok M, Castellino FJ (2001) Structure-function relationships of the NMDA receptor antagonist conantokin peptides. Curr Drug Targets 2: 313-322. doi:10.2174/1389450013348542. PubMed: 11554555. 11554555

[30] Warder SE, Chen ZG, Zhu Y, Prorok M, Castellino FJ et al. (1997) The NMR solution structure of the NMDA receptor antagonist, conantokin-T, in the absence of divalent metal ions. FEBS Lett 411: 19-26. doi:10.1016/S0014-5793(97)00573-5. PubMed: 9247135. 9247135

[31] Warder SE, Prorok M, Chen ZG, Li LP, Zhu Y et al. (1998) The roles of individual gamma-carboxyglutamate residues in the solution structure and cation-dependent properties of conantokin-T. J Biol Chem 273: 7512-7522. doi:10.1074/jbc.273.13.7512. PubMed: 9516452. 9516452

[32] Chen ZG, Blandl T, Prorok M, Warder SE, Li LP et al. (1998) Conformational changes in conantokin-G induced upon binding of calcium and magnesium as revealed by NMR structural analysis. J Biol Chem 273: 16248-16258. doi:10.1074/jbc.273.26.16248. PubMed: 9632684. 9632684

[33] Cnudde SE, Prorok M, Dai Q, Castellino FJ, Geiger JH (2007) The crystal structures of the calcium-bound con-G and con-T[K7gamma] dimeric peptides demonstrate a metal-dependent helix-forming motif. J Am Chem Soc 129: 1586-1593. doi:10.1021/ja065722q. PubMed: 17243678. 17243678

[34] Bliss TV, Lomo T (1973) Long-lasting potentiation of synaptic transmission in the dentate area of the anaesthetized rabbit following stimulation of the perforant path. J Physiol 232: 331-356. PubMed: 4727084. 4727084 10.1113/jphysiol.1973.sp010273 PMC1350458

[35] Alex AB, Baucum AJ, Wilcox KS (2006) Effect of Conantokin G on NMDA receptor-mediated spontaneous EPSCs in cultured cortical neurons. J Neurophysiol 96: 1084-1092. doi:10.1152/jn.01325.2005. PubMed: 16760339. 16760339

[36] Martel MA, Wyllie DJ, Hardingham GE (2009) In developing hippocampal neurons, NR2B-containing N-methyl-D-aspartate receptors (NMDARs) can mediate signaling to neuronal survival and synaptic potentiation, as well as neuronal death. Neuroscience 158: 334-343. doi:10.1016/j.neuroscience.2008.01.080. PubMed: 18378405. 18378405 PMC2635533

[37] Furukawa H, Singh SK, Mancusso R, Gouaux E (2005) Subunit arrangement and function in NMDA receptors. Nature 438: 185-192. doi:10.1038/nature04089. PubMed: 16281028. 16281028

[38] Burnashev N, Schoepfer R, Monyer H, Ruppersberg JP, Günther W et al. (1992) Control by asparagine residues of calcium permeability and magnesium blockade in the NMDA receptor. Science 257: 1415-1419. doi:10.1126/science.1382314. PubMed: 1382314. 1382314

[39] El Gaamouch F, Buisson A, Moustié O, Lemieux M, Labrecque S et al. (2012) Interaction between alphaCaMKII and GluN2B controls ERK-dependent plasticity. J Neurosci 32: 10767-10779. doi:10.1523/JNEUROSCI.5622-11.2012. PubMed: 22855824. 22855824 PMC6621385

[40] Miao Y, Dong LD, Chen J, Hu XC, Yang XL et al. (2012) Involvement of calpain/p35-p25/Cdk5/NMDAR signaling pathway in glutamate-induced neurotoxicity in cultured rat retinal neurons. PLOS ONE 7: e42318. doi:10.1371/journal.pone.0042318. PubMed: 22870316. 22870316 PMC3411656

[41] Auberson YP, Allgeier H, Bischoff S, Lingenhoehl K, Moretti R et al. (2002) 5-phosphonomethylquinoxalinediones as competitive NMDA receptor antagonists with a preference for the human 1A/2A, rather than 1A/2B receptor composition. Bioorg Med Chem Lett 12: 1099-1102. doi:10.1016/S0960-894X(02)00074-4. PubMed: 11909726. 11909726

[42] Bading H, Greenberg ME (1991) Stimulation of protein tyrosine phosphorylation by NMDA receptor activation. Science 253: 912-914. doi:10.1126/science.1715095. PubMed: 1715095. 1715095

[43] Marshall J, Dolan BM, Garcia EP, Sathe S, Tang X et al. (2003) Calcium channel and NMDA receptor activities differentially regulate nuclear C/EBPbeta levels to control neuronal survival. Neuron 39: 625-639. doi:10.1016/S0896-6273(03)00496-3. PubMed: 12925277. 12925277

[44] Balsara R, Li N, Weber-Adrian D, Huang L, Castellino FJ (2012) Opposing action of conantokin-G on synaptically and extrasynaptically-activated NMDA receptors. Neuropharmacology 62: 2227-2238. doi:10.1016/j.neuropharm.2012.01.018. PubMed: 22306487. 22306487 PMC3680358

[45] Klein RC, Siarey RJ, Caruso A, Rapoport SI, Castellino FJ et al. (2001) Increased expression of NR2A subunit does not alter NMDA-evoked responses in cultured fetal trisomy 16 mouse hippocampal neurons. J Neurochem 76: 1663-1669. doi:10.1046/j.1471-4159.2001.00170.x. PubMed: 11259484. 11259484

[46] Klein RC, Warder SE, Galdzicki Z, Castellino FJ, Prorok M (2001) Kinetic and mechanistic characterization of NMDA receptor antagonism by replacement and truncation variants of the conantokin peptides. Neuropharmacology 41: 801-810. doi:10.1016/S0028-3908(01)00119-8. PubMed: 11684144. 11684144

[47] Sheng Z, Dai Q, Prorok M, Castellino FJ (2007) Subtype-selective antagonism of N-methyl-D-aspartate receptor ion channels by synthetic conantokin peptides. Neuropharmacology 53: 145-156. doi:10.1016/j.neuropharm.2007.04.016. PubMed: 17588620. 17588620 PMC3965200

[48] Mallon AP, Auberson YP, Stone TW (2005) Selective subunit antagonists suggest an inhibitory relationship between NR2B and NR2A-subunit containing N-methyl-D: -aspartate receptors in hippocampal slices. Exp Brain Res 162: 374-383. doi:10.1007/s00221-004-2193-6. PubMed: 15580338. 15580338

[49] Hardingham GE, Bading H (2002) Coupling of extrasynaptic NMDA receptors to a CREB shut-off pathway is developmentally regulated. Biochim Biophys Acta 1600: 148-153. doi:10.1016/S1570-9639(02)00455-7. PubMed: 12445470. 12445470

[50] Martel MA, Ryan TJ, Bell KF, Fowler JH, McMahon A et al. (2012) The subtype of GluN2 C-terminal domain determines the response to excitotoxic insults. Neuron 73: 543-556. PubMed: 22578505. 10.1016/j.neuron.2012.03.021 PMC3398391 22578505

[51] Gowd KH, Han TS, Twede V, Gajewiak J, Smith MD et al. (2012) Conantokins derived from the Asprella clade impart conRl-B, an N-methyl d-aspartate receptor antagonist with a unique selectivity profile for NR2B subunits. Biochemistry 51: 4685-4692. doi:10.1021/bi300055n. PubMed: 22594498. 22594498 PMC4153739

[52] Platt RJ, Han TS, Green BR, Smith MD, Skalicky J et al. (2012) Stapling mimics noncovalent interactions of γ-carboxyglutamates in conantokins, peptidic antagonists of N-methyl-D-aspartic acid receptors. J Biol Chem 287: 20727-20736. doi:10.1074/jbc.M112.350462. PubMed: 22518838. 22518838 PMC3370255

